# Comparison of the sensitization profile between children and adults allergic to multiple plant-foods

**DOI:** 10.1186/2045-7022-3-S3-O4

**Published:** 2013-07-25

**Authors:** M Pascal, J Bartra, R Jiménez-Feijoo, J Milà, L Millan, MM Folqué, J Sánchez-López, A Valero, M Juan, R Vilella, AM Plaza, J Yagüe

**Affiliations:** 1 Immunology, Hospital Clinic, Barcelona, Spain; 2 Unitat Al.lèrgia, Servei de Pneumologia, ICT, Hospital Clínic de Barcelona, Barcelona, Spain; 3 Immunoal.lèrgia, Hospital Sant Joan de Déu, Esplugues de Llobregat, Spain

## Background

Component resolved diagnosis (CRD) in a multiplex format is a useful tool for evaluating the panorama of sensitization of polysensitized patients and elucidating the nature of proteins triggering allergic reactions. The aim was to analyze the panorama of sensitization in children and adults allergic to multiple plant-foods and to compare their sensitization profiles.

## Methods

A retrospective, observational and descriptive study of the sensitization profile of 2 cohorts of polysensitized patients (children and adults) from the same geographical area derived to our lab for multiplexed-CRD (ImmunoCAP ISAC, Phadia-ThermoFisher Scientific) since January 2012. All patients had been derived under the same clinical criteria: diagnosis of plant-food allergy to at least 2 non-taxonomically related plant-foods (with clinical history of allergic reaction, positive skin prick test and/or specific IgE to culprit foods).

## Results

The microarray results of 83 children (median age [range] years-old: 11 [3-18]) and 44 adults (32 [20-61]) were analyzed. In children, the panorama of sensitization to plant-food allergens showed a high prevalence of storage proteins of nuts and seeds (80.7%), especially walnut (Jug r 1, Jug r 2) and peanut (Ara h 2, Ara h 6) components. Sensitization to storage proteins occurred with concomitant sensitization to panallergens, mainly LTP (Lipid Transfer Protein, 77.1%), profilins (18%) and/or PR-10 (14.5%), but also without sensitization to cross-reactivity molecules (15.7%). In contrast, in adults the panorama of plant-food sensitization was restricted to LTP (88.6%), with occasional sensitization to storage proteins (13.6%, mainly Jug r 1 and/or Jug r 2), profilins (15.9%) and PR-10 (6.8%). Regarding LTP, most children and adults showed sensitization to almost all LTPs of the microarray. In patients showing sensitization to only one LTP, that was Jug r 3 (3 children and 3 adults), or Pru p 3 (5 children and 1 adult).

## Conclusion

The panorama of sensitization between children and adults, recruited following the same clinical criteria, differs significantly on storage proteins, whereas the high prevalence and broad LTP sensitization profile seems to be similar. Although prospective studies are required, LTP sensitization may start early in life and persist until adulthood. The high prevalence of LTP sensitization is consistent with a Mediterranean area, but the high prevalence of storage proteins observed in children has to be taken into account.

## Disclosure of interest

None declared.

